# Up-regulation of *GLI1* in vincristine-resistant rhabdomyosarcoma and Ewing sarcoma

**DOI:** 10.1186/s12885-020-06985-0

**Published:** 2020-06-03

**Authors:** Joon Won Yoon, Marilyn Lamm, Christopher Chandler, Philip Iannaccone, David Walterhouse

**Affiliations:** 1 Department of Pediatrics, Division of Hematology/Oncology, Ann & Robert H. Lurie Children’s Hospital of Chicago, Northwestern University Feinberg School of Medicine Chicago, Box 30, 225 East Chicago Ave., Chicago, IL 60611 USA; 2 Department of Pathology, Ann & Robert H. Lurie Children’s Hospital of Chicago, Northwestern University Feinberg School of Medicine Chicago, Chicago, IL 60611 USA

**Keywords:** Rhabdomyosarcoma, Ewing sarcoma, Hedgehog pathway, GLI1, Vincristine, Drug resistance

## Abstract

**Background:**

The clinical significance of *GLI1* expression either through canonical Hedgehog signal transduction or through non-canonical mechanisms in rhabdomyosarcoma (RMS) or Ewing sarcoma (EWS) is incompletely understood. We tested a role for Hedgehog (HH) signal transduction and *GL11* expression in development of vincristine (VCR) resistance in RMS and EWS.

**Methods:**

We characterized baseline expression and activity of HH pathway components in 5 RMS (RD, Rh18, Ruch-2, Rh30, and Rh41) and 5 EWS (CHLA9, CHLA10, TC32, CHLA258, and TC71) cell lines. We then established VCR-resistant RMS and EWS cell lines by exposing cells to serially increasing concentrations of VCR and determining the IC_50_. We defined resistance as a ≥ 30-fold increase in IC_50_ compared with parental cells. We determined changes in gene expression in the VCR-resistant cells compared with parental cells using an 86-gene cancer drug resistance array that included *GLI1* and tested the effect of GLI1 inhibition with GANT61 or *GLI1* siRNA on VCR resistance.

**Results:**

We found evidence for HH pathway activity and *GLI1* expression in RMS and EWS cell lines at baseline, and evidence that GLI1 contributes to survival and proliferation of these sarcoma cells. We were able to establish 4 VCR-resistant cell lines (Ruch-2VR, Rh30VR, Rh41VR, and TC71VR). *GLI1* was significantly up-regulated in the Rh30VR, Rh41VR, and TC71VR cells. The only other gene in the drug resistance panel that was significantly up-regulated in each of these VCR-resistant cell lines compared with their corresponding parental cells was the GLI1 direct target and multidrug resistance gene, ATP-binding cassette sub-family B member 1 (*MDR1*). We established major vault protein (*MVP*), which was up-regulated in both vincristine-resistant alveolar RMS cell lines (Rh30VR and Rh41VR), as another direct target of GLI1 during development of drug resistance. Treatment of the VCR-resistant cell lines with the small molecule inhibitor GANT61 or *GLI1* siRNA together with VCR significantly decreased cell viability at doses that did not reduce viability individually.

**Conclusions:**

These experiments demonstrate that *GLI1* up-regulation contributes to VCR resistance in RMS and EWS cell lines and suggest that targeting GLI1 may benefit patients with RMS or EWS by reducing multidrug resistance.

## Background

Children and young adults with recurrent rhabdomyosarcoma (RMS) or Ewing sarcoma (EWS) fare poorly [1–4]. Therefore, mechanisms of drug resistance, which contribute to recurrence need to be fully understood so that effective therapeutic approaches can be established to prevent or reverse development of drug resistance.

The Hedgehog (HH) signal transduction pathway functions during normal development and in cancers [5–8]. The canonical HH signaling pathway is activated when a HH family ligand (Sonic hedgehog [SHH], Indian hedgehog [IHH], or Desert hedgehog [DHH]) interacts with a cell surface receptor in the Patched (PTCH) family (PTCH1 or PTCH2). The interaction between HH and PTCH proteins relieves PTCH-mediated inhibition of the activity of the G protein-coupled seven-span transmembrane protein, Smoothened (SMO). Activation of SMO leads to translocation of GLI family transcription factors (GLI1, GLI2, and GLI3) from cytoplasmic microtubules to the nucleus and transcriptional regulation of target genes. *GLI1* and *PTCH1* are transcriptional targets of HH signaling and their expression serves as an indicator of pathway activation [9, 10]. Non-canonical *GLI1* activation that does not depend on HH, PTCH or SMO, has also been described [11, 12].

In cancer, HH signaling has been implicated in tumorigenicity, cancer stem cell biology, tumor/stromal interactions, and metastasis [13]. In addition, in a wide variety of cancers, including basal cell carcinoma, diffuse large B-cell lymphoma, gliomas, melanoma, myeloid leukemia, and carcinomas of the cervix, colon, esophagus, head/neck, lung, stomach, ovary and prostate, HH signaling has been implicated in the development of resistance to a variety of cytotoxic chemotherapeutic and targeted agents, multidrug resistance, or radiation resistance [14–27].

HH signal transduction pathway components, including HH ligands, PTCH1, SMO, GLI1, GLI2 or GLI3 are present in RMS and EWS cell lines and patient samples [28–36]. The molecular mechanisms that drive HH pathway activation in RMS are incompletely understood [34]. In embryonal RMS (ERMS), there is evidence that HH pathway deregulation sometimes occurs based on loss of heterozygosity at loci for negative regulators of the pathway, including *PTCH1* or Suppressor of Fused (*SUFU*) and based on development of ERMS in *Ptc*+/− or *Sufu*+/− knockout mouse models [33, 37–40]. Gain of 12q13, the *GLI1* locus, has been reported more commonly in alveolar RMS (ARMS) [41, 42]. In EWS, *GLI1* has been shown to be a direct transcriptional target of the EWSR1-FLI1 fusion-protein, which is found in the majority of EWS cases [35, 36, 43, 44].

The clinical significance of *GLI1* activation either through canonical or non-canonical mechanisms is incompletely understood in RMS and EWS. Indeed, debate continues whether markers of HH signaling are present in higher levels in ERMS or ARMS and whether activation of HH signaling correlates with patient outcome [30, 45]. Therefore, we tested the role of HH signal transduction and *GLI1* expression in development of a multidrug resistance phenotype in RMS and EWS by establishing vincristine (VCR)-resistant cells.

## Methods

### RMS and EWS cell lines

We obtained RD cells from ATCC (Manassas, VA). Rh18, Rh30, and Rh41 cells were obtained from Dr. Houghton, Ruch-2 cells from Dr. Schäfer, and UKF-Rhb-1 cells from Dr. Cinatl Jr. We obtained CHLA9, CHLA10, TC32, CHLA258 and TC71 from the Children’s Oncology Group. All cells were cultured in media supplemented with 10–20% fetal bovine serum, 100 U/ml penicillin, and 100 μg/ml streptomycin (Thermo Fisher, MA).

### Reverse transcriptase polymerase chain reaction (RT PCR)

We isolated total RNA from the cell lines using the Qiagen RNeasy mini kit (Qiagen, Valencia, CA). We performed RT PCR using the One-Step RT PCR kit (Qiagen, Valencia, CA) or TaqMan Gene Expression Assay reagents (Applied Biosystems, Foster City, CA). We completed 30–35 cycles of PCR, including denaturation for 30 s, annealing for 30 s, and amplification for 1 min. The following primers were used for PCR: *DHH* sense 5′-GCTCTCCTGACCAATCTACTG-3′ and *DHH* antisense 5′-TCGTGCCCAACTACAACCC-3′, *IHH* sense 5′-CAAGCAGTTCAGCCCCAATG-3′ and *IHH* antisense 5′-CTGGTTCATCACCGAGATAGCC-3′, *SHH* sense 5′-CAGAGGTGTAAGGACAAGTTGAACG-3′ and *SHH* antisense 5′-AAAGTGAGGAAGTCGCTGTAGAGC-3′, *PTCH1* sense 5′-CCTGGACGACATCCTGAAATCC-3′ and *PTCH1* antisense 5′-GCGAGAAATGGCAAAACCTGAG-3′, *SMO* sense 5′-TGGCTTTGTGCTCATTACCTTCAG-3′ and *SMO* antisense 5′-ATCCGCTTTGGCTCATCGTC-3′, *GLI1* sense 5′-AGTCATACTCACGCCTCGAA-3′ and *GLI1* antisense 5′-GACCATGCACTGTCTTGACA-3′, *GLI2* sense 5′-AAGGATTGCCACCCAGGACG-3′ and *GLI2* antisense 5′-CCGACTCACTGCTCTGCTTGTT-3′, *GLI3* sense 5′-CGAACAGATGTGAGCGAGAAAGC-3′ and *GLI3* antisense 5′-AAAGATGAGGAGGGTGGTAGTGGG-3′, *PAX3-FOX01* sense 5′-CCGACAGCAGCTCTGCCATC-3′ and *PAX3-FOX01* antisense 5′-ATGAACTTGCTGTGTAGGGACAG-3′, *EWSR1-FLI1* sense 5′- GCACCTCCATCCTACCCTCCT − 3′ and *EWSR1-FLI1* antisense 5′- CTTACTGATCGTTTGTGCCCC-3′(long) or *EWSR1-FL1I* antisense 5′- TGGCAGTGGGTGGGTCTTCAT-3′(short), and *GAPDH* sense 5′-TGATGACATCAAGAAGGTGGTGAAG-3′ and *GAPDH* antisense 5′-TCCTTGGAGGCCATGTGGGCCAT-3′.

### Western blot analysis

We prepared cell lysates using Tris.HCl buffer (pH 7.4), containing 150 mM NaCl, protease inhibitor cocktail (Thermo Fisher, MA), 0.5 mM DTT, and 1% TritonX-100. We loaded 50–100 μg of protein onto 4–15% SDS-PAGE gels (BioRad, Hercules, CA). After electrophoresis, we blotted the proteins onto nitrocellulose membranes (BioRad, Hercules, CA) and probed with polyclonal antibodies against human GLI1 protein (Cell Signaling, Danvers, MA) or against GAPDH (Santa Cruz Biotech, Santa Cruz, CA). We visualized the protein using a chemiluminescence kit (Pierce Inc., Rockford, IL).

### Quantitative RT PCR (qRT PCR)

cDNA was synthesized using a high capacity cDNA reverse transcription kit (Applied Biosystems, Foster City, CA). PCR was performed using TaqMan universal PCR master mix and the following conditions; 50 °C for 2 min, 95 °C for 10 min and 40 cycles of 95 °C for 15 s and 60 °C for 1 min (Applied Biosystems, Foster City, CA). Primers and probes for *GLI1* (Hs00171790_ml), *PTCH1* (Hs00970980_ml), and *GAPDH* (Hs99999905_ml) were purchased from Applied Biosystems (Foster City, CA). The experiments were completed in triplicate and an average and standard deviation were calculated.

### Immunofluorescence for primary cilia

We grew RMS and EWS cells in 8-well chamber slides (Nunc, Rochester, NY). Cells were fixed with 4% paraformaldehyde/0.5% TritonX-100 in PBS for 30 min at room temperature with rocking. Cells were washed again with PBS at room temperature and were then blocked for 1 h with 10% donkey serum. We incubated the cells with anti-acetylated alpha-tubulin (Sigma, St. Louis, MO, 1:5000 dilution), and pericentrin (Abcam, Cambridge, MA, 1:500 dilution) or pericentrin2 (Santa Cruz Biotech, Santa Cruz, CA, 1:500 dilution) antibodies overnight at 4 °C. Cells were washed with PBS and incubated with the secondary antibodies at 1:300 dilution (donkey anti-goat IgG Alexa488, [Alexa, Eugene, OR], or donkey anti-mouse IgG Alexa568, [Alexa, Eugene, OR]) for 1 h at room temperature. The nuclei were stained with 4′,6-diamidino-2-phenyindole (DAPI) at 1:2000 dilution (Biotium, Hayward, CA) for 20 min at room temperature with rocking. Immunofluorescence was observed using a Zeiss LSM510 or LSM880 META confocal laser scanning microscope.

### Treatment of cells with hedgehog ligands

We exposed RMS and EWS cells to 1 μg/ml of DHH, IHH, or SHH peptide (R&D Systems, Minneapolis, MN) for 24 h at 37 °C in 5% CO_2_ with serum-free culture media.

### Treatment of cells with SMO or GLI1 inhibitors and/or VCR

We added 2500–5000 cells in 0.1 ml of culture media supplemented with 10–20% (v/v) fetal bovine serum to Falcon 96-well cell culture plates (Becton Dickinson, Franklin Lakes, NJ) and cultured overnight. We then added 0–10 μM vismodegib (LC Lab, Woburn, MA), 0–40 μM GANT61 (Sigma, St. Louis, MO), 0–8 nM *GLI1* siRNA (IDT, Coralville, IA), and/or 0–50 nM VCR (LC Lab, Woburn, MA) or equal volumes of solvent (ethanol or DMSO). We used 1 μl of interferon (Polyplus, NY) for *GLI1* siRNA transfection. Cells were incubated with the inhibitor and/or VCR at 37 °C with 5% carbon dioxide for up to 72 h.

### Methylthiazolyl diphenyl-tetrazolium bromide (MTT) assays

We added 10 μl of MTT reagent (5 mg/ml MTT in 1X PBS) to 0.1 ml culture media containing the cells. The mixture was incubated for 3 h at 37 °C, then we added 0.1 ml of solubilization solution (10% SDS in 0.01 M HCl) and incubated the mixture overnight at 37 °C. Absorbances at 570 and 650 nm were measured using a Ceres UV900H Di ELISA plate reader (Bio-Tek Instruments, Inc., Highland Park, VT). Background readings at 650 nm were subtracted from optical density readings at 570 nm. The experiments were completed in triplicate and an average and standard deviation calculated.

### 5-bromo-2′-deoxyuridine (BrdU) assays

We measured cell proliferation using a BrdU cell proliferation assay kit (MilliporeSigma, Burlington, MA). Cells were incubated with BrdU for 1 h at 37 °C following incubation for 24 h. Cells were fixed and BrdU incorporation was detected with anti-BrdU detector antibody. The signal was measured using a Ceres UV900H Di ELISA plate reader (Bio-Tek Instruments, Inc., Highland Park, VT). The experiments were completed in triplicate and an average and standard deviation calculated.

### Caspase 3/7 assays

We measured apoptosis using a caspase 3/7 assay kit (Promega, Madison, WI). Cells were incubated with an equal volume of 2X caspase assay solution for 1 h at room temperature in the dark. Caspase activity was measured with a luminometer (Berthold, Oak Ridge, TN). The experiments were completed in triplicate and an average and standard deviation calculated.

### Preparation of VCR-resistant cell lines

We established VCR-resistant RMS and EWS cell lines by exposing cells to serially increasing concentrations of VCR. We defined resistance as a 30-fold increase in the IC_50_ compared with parental cells before exposure to VCR together with persistence of resistance over several passages.

### Cancer drug resistance array

qRT PCR using an 86-gene cancer drug resistance PCR array (Qiagen, Germantown, MD) was performed as described in the manual. The commercial array was modified to add *GLI1* and the direct GLI1 target, ATP binding cassette subfamily B member, transporter 1 (*TAP1*) to the commercially available 84-gene panel. Total RNA was purified from the parental and VCR-resistant RMS cells using an RNAeasy mini kit (Qiagen, Valencia, CA). Genomic DNA was removed using on-column DNase I treatment. RNA was quantified using a Nanodrop Spectrophotometer (Thermo Scientific). Reverse transcription was carried out using 1 μg of total RNA and the RT^2^ First Strand kit (Qiagen). Real-Time PCR was carried out using a 7500 fast PCR machine (Applied Biosystems) as described in manufacturer’s protocol. The threshold cycle (Ct) of each well was calculated using the instrument’s software and the fold change was calculated by comparing the Ct of the parental cell line and the corresponding VCR-resistant cell line for a given gene. Any gene with a +/− 2-fold change in expression in the VCR-resistant cells compared with the corresponding parental cells was selected for further analysis. The results were shown as means and standard deviations. Statistical significance was calculated using a Student t-test and a *p* ≤ 0.05 was considered significant.

### Electrophoretic mobility shift assays

Two microliters of Rh30 cell lysate was mixed with 5 μl of 2X binding buffer (50 mM HEPES, pH 7.5, 50 mM KCl, 5 mM MgCl_2_, 1 mM DTT, 20% glycerol (v/v), 50 mM poly (dI-dC), and 10 mM ZnSO_4_), H_2_O, and 0 or 1 μl (20 pmol) of unlabeled competitor oligonucleotide. The mixture was incubated at 4^o^ C for 10 min. One microliter (155 fmol) of double stranded digoxigenin-labeled probe was added and the mixture was incubated at 4 °C for an additional 20 min. We used the following probes, each of which included a different consensus GLI1 binding site with up to 2 bp mismatches in the *MVP* promoter; *MVP1* sense begins at nucleotide − 1710 in the *MVP* promoter: 5′-CATGTTGGCGAGGCTGGTCTTGAACTCCT-3′ and *MVP1* antisense 5′-AGGAGTTCAAGACCAGCCTCGCCAACATG-3′, *MVP2* sense begins at nucleotide − 505 in the *MVP* promoter: 5′- GTTTTCTATTGAACACCTATAGAGAGAGT − 3′ and *MVP2* antisense 5′- ACTCTCTCTATAGGTGTTCAATAGAAAAC -3′, *MVP3* sense begins at nucleotide − 438 in the *MVP* promoter: 5′- GTTTTCTATTGAACACCTATTCAGAGACC − 3; and *MVP3* antisense 5′- GGTCTCTGAATAGGTGTTCAATAGAAAAC -3′, *MVP4* sense begins at nucleotide − 233 in the *MVP* promoter: 5′- CCATCTCGGGCCCTCCAACTCCTCCCAGTCCCACTCCAG − 3′ and *MVP4* antisense 5′- CTGGAGTGGGACTGGGAGGAGTTGGAGGGCCCGAGATGG − 3′, and *MVP5* sense begins at nucleotide − 172 in the *MVP* promoter: 5′- AGAAACCCATGAGCACTCAGGGAGCAGTG − 3′ and *MVP5* antisense 5′- CACTGCTCCCTGAGTGCTCATGGGTTTCT − 3′. The GLI consensus binding sites are underlined in the probes. The GLI1-*MVP* complexes were separated by electrophoresis, transferred onto nitrocellulose membranes, and bands were visualized by anti-digoxigenin antibody and chemiluminescence (Roche, Mannheim, Germany).

### Chromatin immunoprecipitation (ChIP)

ChIP was performed, using Rh41 and Ruch-2 cells and the ChIP-IT express chromatin immunoprecipitation kit (Active Motif, Carlsbad, CA). DNA and protein were cross-linked with formaldehyde for 10 min at room temperature. The DNA-protein complexes were sheared by sonication, the cell lysate was cleared by centrifugation at 14,000 x g for 10 min, and anti-c-terminal-GLI1 antibody was added (Santa Cruz Biotech, Santa Cruz, CA). Antibody-Protein-DNA complexes were precipitated with protein G magnetic beads. DNA was purified and used for PCR amplification of GLI1 binding sites. We used the following primers: *MVP 4,5* sense 5′- CCTCCTGGGTTGAAGCGATT − 3′ and *MVP 4,5* antisense 5′- TGCTCTTCCCTGGCAAGATG − 3′.

### Co-transfection assays

We co-transfected HeLa cells with 0–1000 ng of pCMV-*GLI1* effector plasmid, 200 ng of pMVP407 reporter construct (obtained from Dr. Furukawa, Kagoshima University), and 20 ng of Renilla control reporter DNA (Promega, Madison, WI), using 2–6 μl of Lipofectamine 2000 reagent (Gibco-BRL, Grand Island, NY). A total of 3 μg of DNA was transfected in each experiment and the difference was made up with pUC18 carrier DNA. Cell lysates were prepared 24 h after transfection. Twenty microliters of cell lysate was assayed by adding 100 μl of substrate solution (Promega, Madison, WI). The experiments were performed at least in triplicate and results expressed as an average with standard deviation.

### Statistical analysis

Differences between groups were assessed using a Student’s t-test unless stated otherwise. *p* ≤ 0.05 was considered significant.

## Results

### RMS and EWS cell lines have active HH signal transduction pathways

**Fig. 1:**
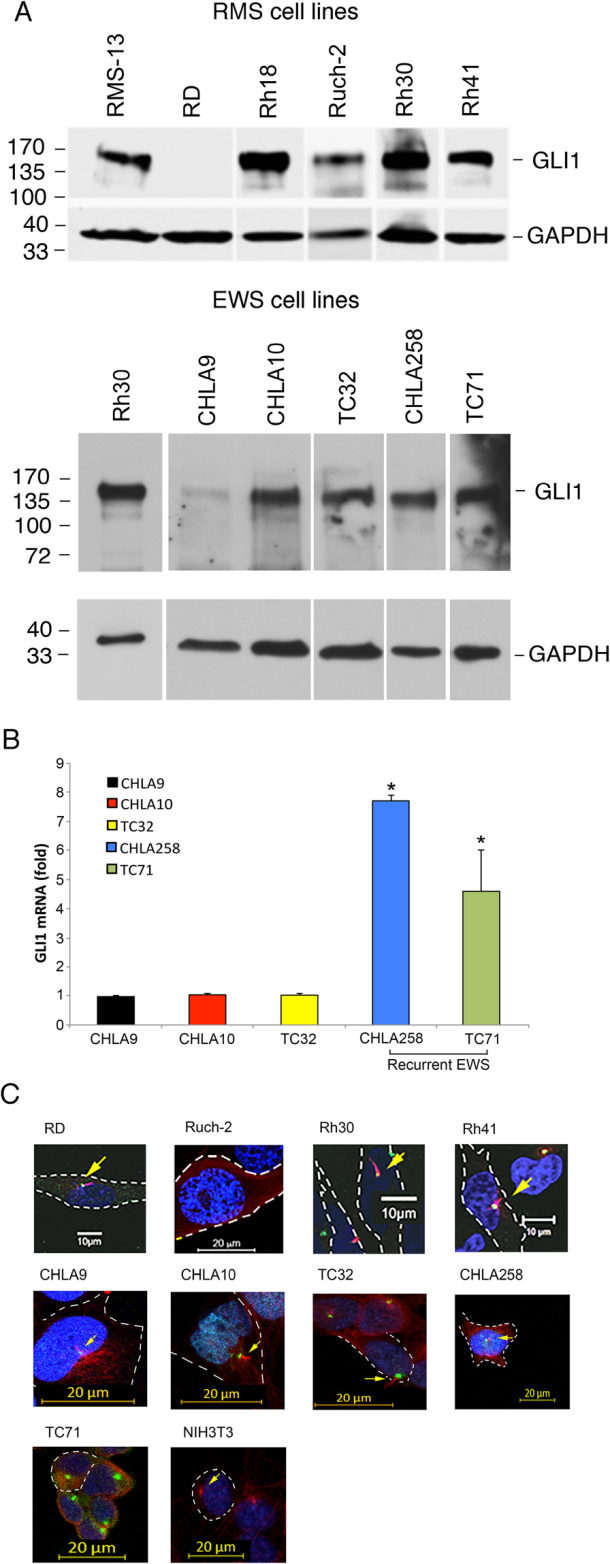
*GLI1* expression and primary cilia in RMS and EWS cell lines. **a** Expression of GLI1 protein by Western blot in RMS and EWS cell lines. Size markers in kilodaltons (kD) are included. *GLI1*-amplified RMS-13 rhabdomyosarcoma cells or *GLI1*- amplified Rh30 cells were used as positive controls. The GAPDH control was included to show comparability of protein loading among lanes. The blots have been cropped and full-length blots are presented in Supplementary Figure 1A. **b** EWS cell lines that were established following recurrence (CHLA258 and TC71) had significantly higher *GLI1* expression compared with cell lines established at the time of diagnosis (CHLA9, CHLA10, and TC32) by qRT PCR. **c** Immunofluorescence microscopy for primary cilia in RMS and EWS cell lines. Cell line names are indicated above the images. Green = pericentrin or pericentrin2 (centriole component), red = acetylated alpha tubulin (primary cilia component), and blue = DAPI nuclear staining. Primary cilia, indicated by arrows, were seen in RD, Rh30, Rh41, CHLA9, CHLA10, CHLA258, and TC32 cells. NIH3T3 cells were used as a positive control. White dotted lines indicate cell borders. Scale bars are shown. Microsoft Exel and Adobe Photoshop were used to prepare Figure 1

We determined the expression of HH pathway components in three ERMS cell lines (RD, Rh18, and Ruch-2), two PAX3-FOX01 fusion-positive ARMS cell lines (Rh30 and Rh41), and five EWSR1-FLI1 fusion-positive EWS cell lines (CHLA9, CHLA10, TC32, CHLA258, and TC71) by RT PCR (Table 1). All of the RMS cell lines expressed *IHH*, *PTCH1*, *SMO*, *GLI1*, *GLI2*, and *GLI3*. We demonstrated GLI1 protein in Rh18, Ruch-2, Rh30, and Rh41 cells but not RD cells by Western blot (Fig. 1a). We did not find inactivating mutations in exons 1–24 of *PTCH1* in any of the RMS cell lines that we tested (RD, Rh18, Rh30 or Rh41) that could contribute to HH pathway activation and expression of *GLI1* (data not shown). All of the EWS cell lines expressed *PTCH1*, *SMO*, *GLI1*, and *GLI3* by RT PCR and GLI1 protein by Western blot (Table 1 and Fig. 1a). We demonstrated significantly higher expression of *GLI1* by qRT PCR in CHLA258 and TC71 EWS cells, which were both established following chemotherapy at the time of recurrence compared with CHLA9 cells, which were established prior to any therapy (Fig. 1b). Two other non-recurrent EWS cell lines, CHLA10 and TC32, also had low *GLI1* expression.

**Table 1:** RNA expression of HH pathway components by RT PCR in RMS and EWS cell lines

	DHH	IHH	SHH	PTCH1	SMO	GLI1	GLI2	GLI3	PAX3-FOX1	EWSR1-FLI1
RD	+	+	–	+	+	+	+	+	–	NA
Rh18	+	+	–	+	+	+	+	+	–	NA
Ruch-2	–	+	–	+	+	+	+	+	–	NA
Rh30	–	+	–	+	+	+	+	+	+	NA
Rh41	–	+	–	+	+	+	+	+	+	NA
CHLA9	–	–	–	+	+	+	–	+	NA	+
CHLA10	–	–	+	+	+	+	–	+	NA	+
TC32	–	–	–	+	+	+	–	+	NA	+
CHLA258	–	–	–	+	+	+	–	+	NA	+
TC71	–	–	–	+	+	+	–	+	NA	+

Since HH signaling occurs in primary cilia, we used immunofluorescence for acetylated alpha-tubulin and pericentrin or pericentrin2 to determine whether RMS and EWS cells have primary cilia (Fig. 1c). We identified primary cilia in the majority of the RMS (RD, Rh30, and Rh41) and EWS (CHLA9, CHLA10, CHLA258 and TC32) cell lines.

**Fig. 2:**
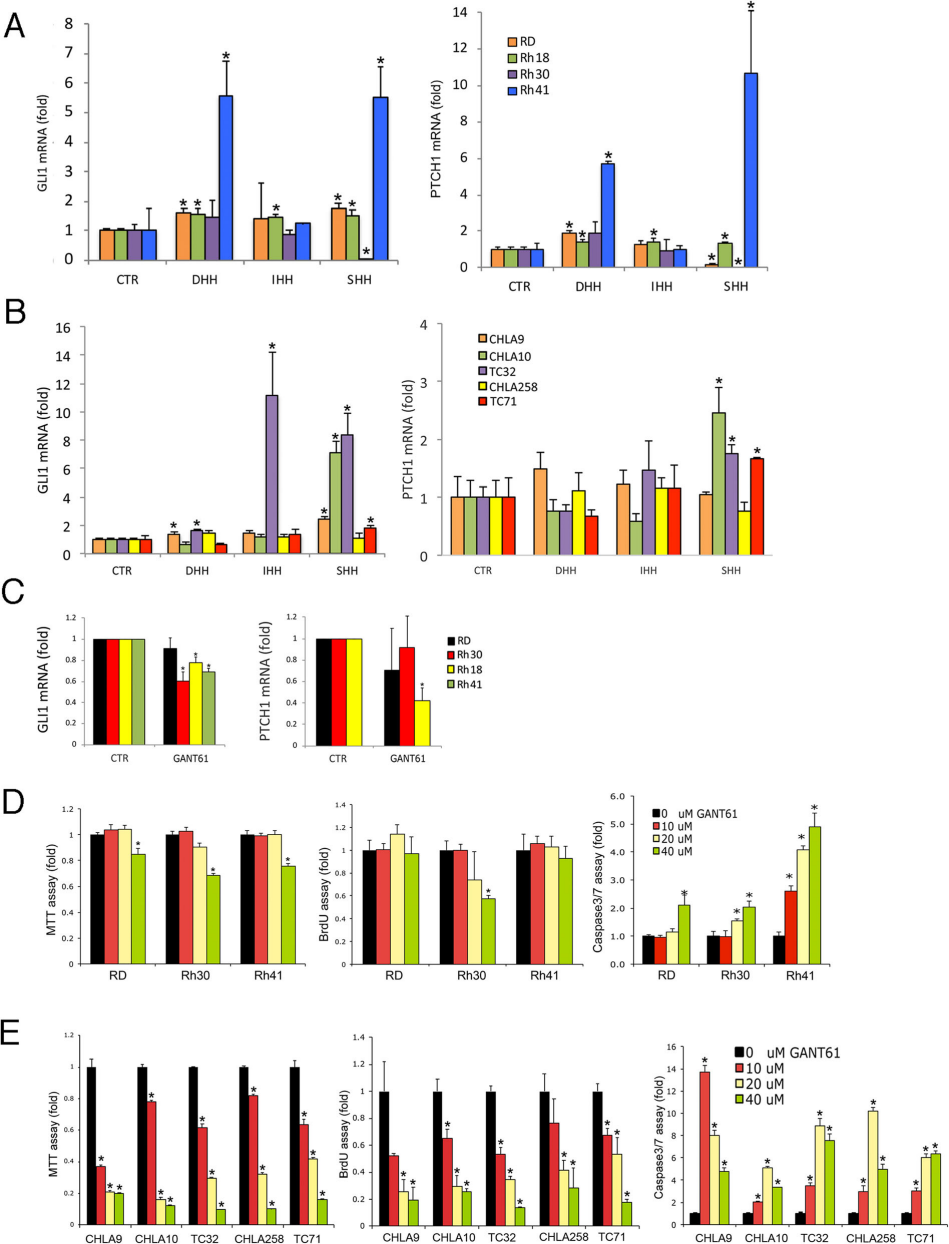
Modulation of HH pathway activity in RMS and EWS cells following exposure to HH ligands or a GLI1 inhibitor. **a***GLI1* (left panel) or *PTCH1* (right panel) mRNA expression was sometimes significantly increased (indicated by *) following exposure to HH ligands in RD, Rh18, and Rh41 cells compared with their corresponding control cells (CTR) that were not treated with HH ligands. The experiments were conducted in triplicate. Averages (bars) and standard deviations (brackets) are shown. **b***GLI1* (left panel) or *PTCH1* (right panel) mRNA expression was sometimes significantly increased (indicated by *) following exposure to HH ligands in CHLA9, CHLA10, TC32, and TC71 cells compared with their corresponding control cells (CTR) that were not treated with HH ligands. The experiments were conducted in triplicate. Averages (bars) and standard deviations (brackets) are shown. **c***GLI1* expression (left) measured by qRT PCR was significantly decreased, indicated by *, followed treatment with GANT61 in Rh30, Rh18, or Rh41 cells. *PTCH1* expression (right) measured by qRT PCR was significantly decreased, indicated by *, following treatment with GANT61 in Rh41 cells. The experiments were conducted in triplicate. Averages (bars) and standard deviations (brackets) are shown. **d** MTT (left panel), BrdU (middle panel), and Caspase 3/7 (right panel) assays in RMS cell lines (listed on the x-axis). Apoptosis increased with GLI1 down-regulation with GANT61 in RMS cells. The experiments were conducted in triplicate. Averages (bars) and standard deviations (brackets) are shown. * = statistically significant difference compared with no GANT61 treatment (black bar). **e** MTT (left panel), BrdU (middle panel), and Caspase 3/7 (right panel) assays in EWS cell lines (listed on the x-axis). Cell viability and cell proliferation decreased and apoptosis increased with GLI1 down-regulation with GANT61 in EWS cells. The experiments were conducted in triplicate. Averages (bars) and standard deviations (brackets) are shown. * = statistically significant difference compared with no GANT61 treatment (black bar). Microsoft Exel and Adobe Photoshop were used to prepare Figure 2

To assess responsiveness of RMS and EWS cells to HH ligands, we exposed RMS and EWS cells to DHH, IHH, and SHH. RD, Rh18, and Rh41 RMS cells showed up-regulation of targets of HH signaling, *GLI1* and/or *PTCH1*, in response to HH ligands (Fig. 2a). We also saw up-regulation of the HH-target genes *GLI1* and/or *PTCH1* in response to HH ligands in CHLA9, CHLA10, TC32 and TC71 EWS cell lines (Fig. 2b).

To assess the effect of HH pathway up-regulation on the biology of RMS and EWS cells, we assessed cell viability (MTT assay), cell proliferation (BrdU assay), and apoptosis (Caspase 3/7 assay) in RMS and EWS cells with and without SHH ligand. Exposure to SHH did not significantly affect the results of these assays (data not shown). To assess the effect of HH pathway/*GLI1* down-regulation on the biology of RMS and EWS cells, we treated the cells with the GLI inhibitor GANT61 (0–40 μM) [46]. GANT61 inhibits GLI1 function and sometimes down-regulates *GLI1* and *PTCH1* expression in RMS cell lines (Fig. 2c). In RMS cells, apoptosis increased in each of the cell lines following treatment with GANT61, suggesting a role for GLI1 in survival of these cells (Fig. 2d). Cell viability or cell proliferation decreased only at the highest GANT61 concentration that we tested. In EWS cells, cell viability and cell proliferation decreased, whereas apoptosis increased in each of the cell lines following treatment with GANT61, suggesting roles for GLI1 in proliferation and survival of EWS cells (Fig. 2e).

### GLI1 is up-regulated in vincristine-resistant RMS and EWS cell lines

To determine if *GLI1* up-regulation occurs as cells develop resistance to VCR, we established VCR-resistant RMS (Ruch-2VR, Rh30VR, and Rh41VR) and EWS (TC71VR) cell lines by exposing parental cells to serially increasing concentrations of VCR. The goal was to increase the IC_50_ ≥ 30-fold (Table 2). We were not able to establish VCR-resistant RD, Rh18, CHLA9, CHLA10, TC32, or CHLA258 cells that met this standard.

**Table 2:** IC_50_ of RMS and EWS cell lines before and after exposure to vincristine

Cell line	Parental IC_50_ (nM)	VCR-resistant IC_50_ (nM)	Fold change
Ruch-2	95	11,320	119
Rh30	5.7	2228	391
Rh41	5.2	1996	384
TC71	22	1000	45

**Fig. 3:**
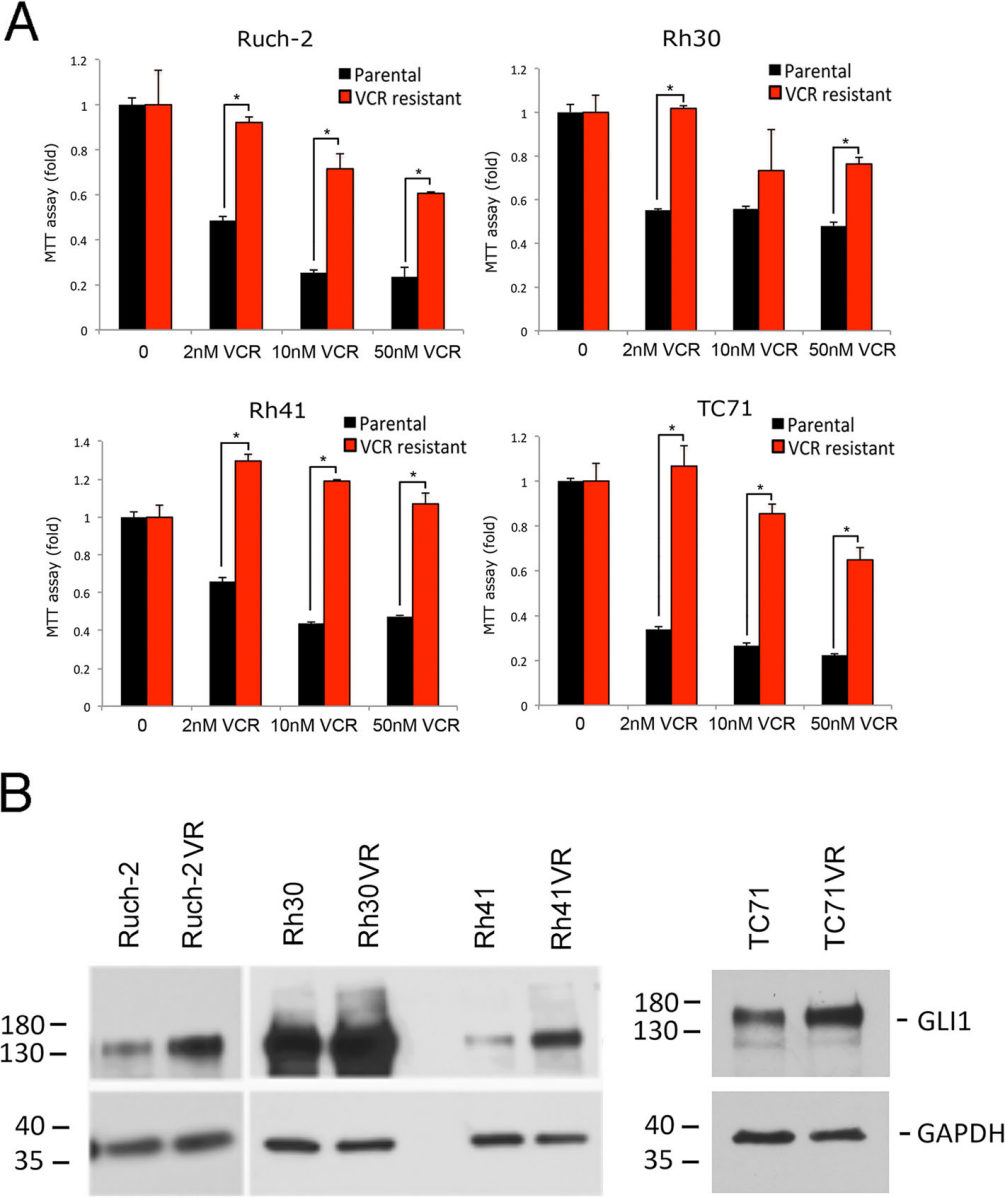
Cell viability and *GLI1* expression in VCR-resistant cell lines. **a** VCR-resistant cell lines retained viability to a greater extent when treated with VCR than their corresponding parental cell lines. MTT assays were conducted in triplicate. Averages (bars) and standard deviations (brackets) are shown. * = statistically significant difference in viability between the parental (black bars) and VCR-resistant (red bars) cell lines at a given VCR concentration. **b** Western blots showed up-regulation of GLI1 in VCR-resistant RMS (Ruch-2VR, Rh30VR, Rh41VR) (left panel) and EWS (TC71VR) (right panel) cell lines compared with their corresponding parental cells (Ruch-2, Rh30, Rh41, TC71). GAPDH was used to indicate equal loading between lanes. Size markers in kD are shown. The blots have been cropped and full-length blots are presented in Supplementary Figure 3B. Microsoft Exel and Adobe Photoshop were used to prepare Figure 3

As expected, we showed that VCR-resistant cells retained viability to a greater degree than their corresponding parental cells when exposed to increasing concentrations of VCR (0–50 nM) (Fig. 3a). The VCR-resistant cell lines exhibited up-regulation of GLI1 protein compared to their respective parental cell lines (Fig. 3b).

**Fig. 4:**
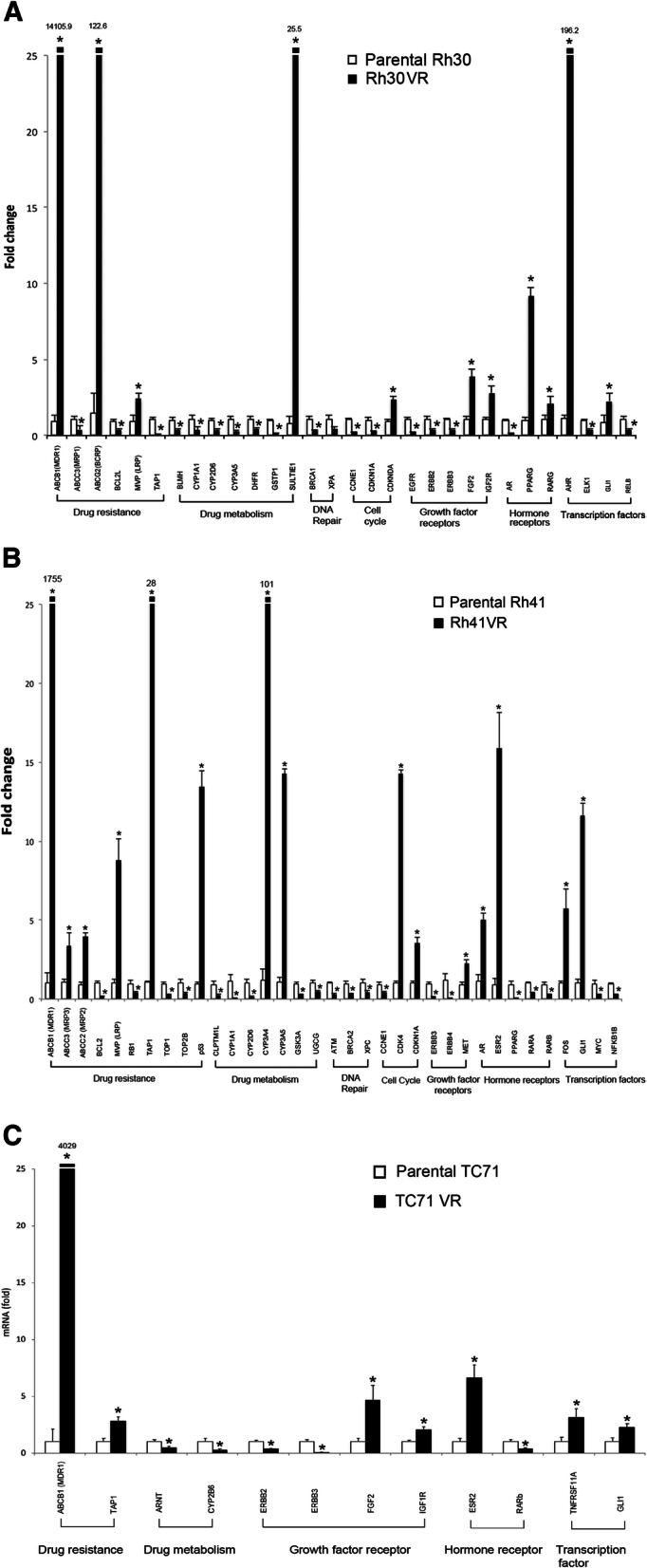
Gene expression changes in VCR-resistant RMS and EWS cells compared with parental cells by PCR array. **a** Rh30VR cells showed significant changes (at least 2-fold up-regulated or down-regulated, and *p* ≤ 0.05) in gene expression in 30 genes, including *GLI1*, that were included in the 86 gene drug resistance panel compared with parental cells. The experiment was completed in triplicate. Averages (bars) and standard deviations (brackets) are shown. * indicates *p* ≤ 0.05 and fold change is indicated on the y-axis. **b** Rh41VR cells showed significant changes (at least 2-fold up-regulated or down-regulated, and *p* ≤ 0.05) in gene expression in 35 genes, including *GLI1*, that were included in the 86 gene drug resistance panel compared with the parental cells. The experiment was completed in triplicate. Averages (bars) and standard deviations (brackets) are shown. * indicates *p* ≤ 0.05 and fold change is indicated on the y-axis. **c** TC71VR cells showed significant changes (at least 2-fold up-regulated or down-regulated, and *p* ≤ 0.05) in gene expression in 12 genes, including *GLI1*, that were included in the 86 gene drug resistance panel compared with the parental cells. The experiment was completed in triplicate. Averages (bars) and standard deviations (brackets) are shown. * indicates *p* ≤ 0.05 and fold change is indicated on the y-axis. Microsoft Exel and Adobe Photoshop were used to prepare Figure 4

To more globally characterize changes in gene expression in the VCR-resistant cells compared with their corresponding parental cells, we used an 86-gene cancer drug resistance array (Qiagen). *GLI1* expression was significantly increased (≥2.0 fold and *p* ≤ 0.05) in VCR-resistant ARMS cell lines compared with their corresponding parental cells (Rh30VR cells: 2.3 fold increased and *p* = 0.04, Rh41VR cells: 10.1 fold increased and *p* = 0.0008) (Fig. 4a and b). In Ruch-2VR ERMS cells, *GLI1* expression was up-regulated (*p* = 0.02) but did not reach the bar of a 2.0-fold increase (1.7 fold increased) (data not shown). To provide data from an additional VCR-resistant ARMS cell line we assessed expression of *GLI1* in parental and VCR-resistant UKF Rhb-1 ARMS cells obtained from Dr. Cinatl (Frankfurt, Germany). Once again, we found significant up-regulation of *GLI1* (2.3 fold and *p* = 0.0007) (data not shown). These results suggest that GLI1 potentially plays a role in the development of a multi-drug resistance phenotype in fusion-positive ARMS.

**Fig. 5:**
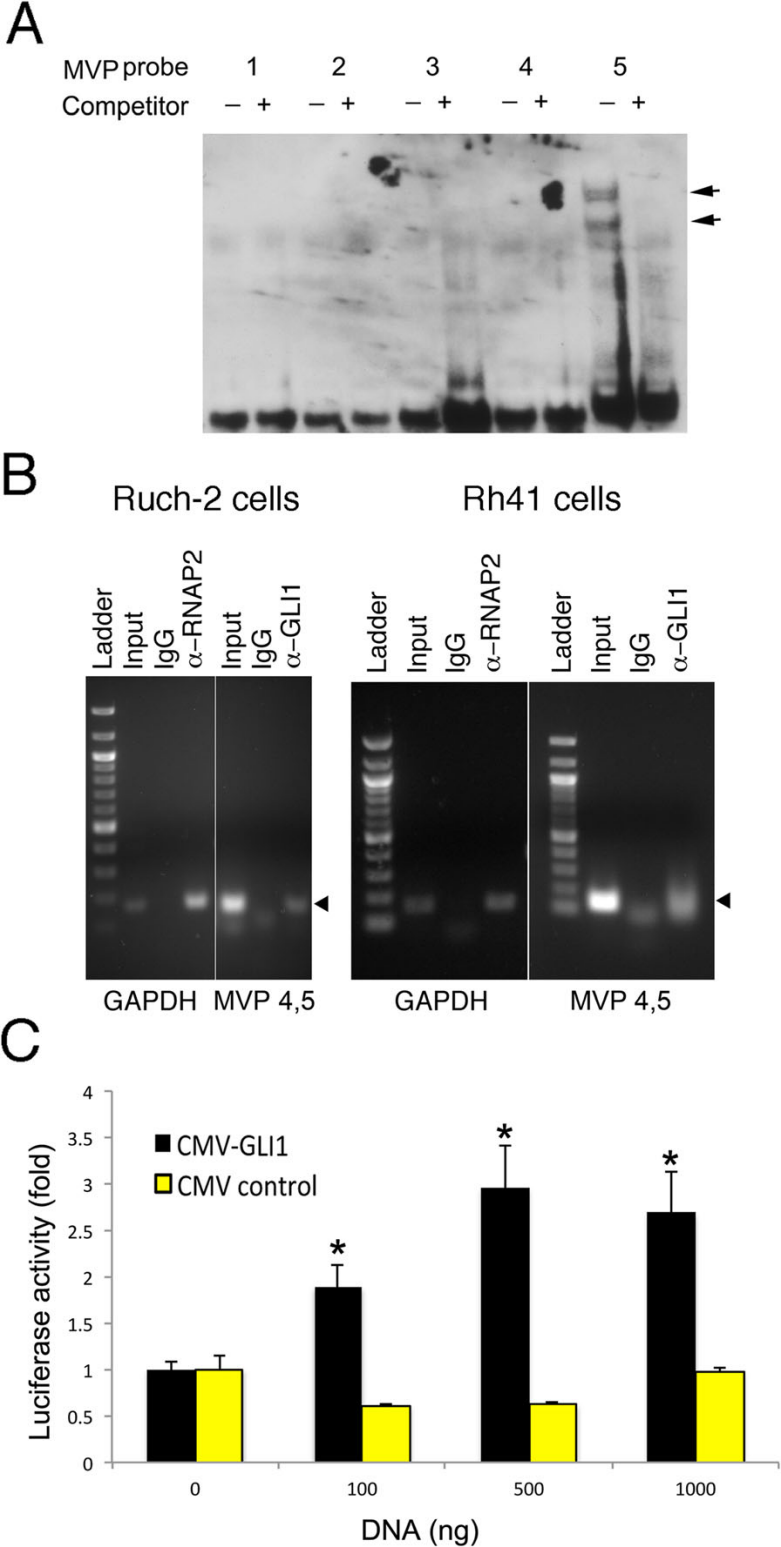
*MVP* is a direct transcriptional target of GLI1. **a** Electrophoretic mobility shift assays were completed using Rh30 cell lysate and 5 different probes that span the *MVP* promoter region and include each of the GLI consensus binding sites. Shifted bands (indicated by arrows) were visualized by anti-digoxigenin antibody and chemiluminescence for *MVP* probe 5 (lane marked -). Cold competitor was used to show specificity of binding in the lanes marked +. The gel has been cropped and the full-length gel is presented in Supplementary Figure 5A. **b** Chromatin immunoprecipitation was performed using Ruch-2 (left panel) and Rh41 (right panel) cells. PCR-amplified DNA bands are indicated in each of the cell lines by the arrows. IgG was used as negative control and anti-RNA polymerase2 (alpha-RNAP2) was used as positive control for the ChIP. Alpha-GLI1 = anti-c-terminal-GLI1 antibody. *MVP* 4,5 = PCR primers spanned the region from *MVP* probe 4 through *MVP* probe 5, which were also used in 5A. The gels have been cropped and full-length gels are presented in Supplementary Figure 5B. **c** Cotransfection assays were completed by transfecting HeLa cells with 0–1000 ng pCMV-*GLI1* (black bars) or pCMV-control (yellow bars) effector plasmids, 200 ng of pMVP407 reporter construct and 20 ng of Renilla control reporter DNA. The experiments were performed in triplicate and results are expressed as an average with standard deviation. * indicates statistically significantly increased reporter activity using a given amount of effector pCMV-*GLI1* DNA compared with control effector DNA (*p* ≤ 0.05). Microsoft Exel and Adobe Photoshop were used to prepare Figure 5

Expression of only 2 additional genes was also significantly up-regulated (≥2 fold and *p* ≤ 0.05) in both the Rh30VR and Rh41VR ARMS cell lines compared with their corresponding parental cells: ATP-binding cassette sub-family B member 1 (*MDR1*) (Rh30 cells: 13,307 fold increased and *p* = 0.002, Rh41 cells: 1755 fold increased and *p* = 0.02) and major vault protein (*MVP*) (Rh30 cells: 2.4 fold increased and *p* = 0.002, Rh41 cells: 8.8 fold increased and *p* = 0.008) (Fig. 4a and b). *MDR1* is a known target of GLI1 [26]. Gel mobility shifts and chromatin immunoprecipitation showed interaction of GLI1 with a consensus GLI binding site in the *MVP* promoter (Fig. 5a and b). Cotransfection assays showed that GLI1 up-regulates reporter gene expression through the *MVP* promoter (Fig. 5c), establishing *MVP* as another target of GLI1 during development of drug resistance. Four genes were significantly down-regulated (≥2 fold and *p* ≤ 0.05) in both the Rh30VR and Rh41VR ARMS cell lines compared with their corresponding parental cells: *CYP1A1* (2.6 fold decreased and *p* = 0.01), *CYP2D6* (2.8 fold decreased and *p* = 0.008), cyclin E1 (*CCNE1*) (4.4 fold decreased and *p* = 0.008), and *ERBB3* (6.8 fold decreased and *p* = 0.006).

*GLI1* was significantly up-regulated (≥2 fold and *p* ≤ 0.05) in TC71VR cells compared with parental cells (2.2 fold increased and *p* = 0.01) together with 6 other genes: *MDR1* (4029 fold increased and *p* = 0.0002), transporter 1, ATP-binding cassette subfamily B member (*TAP1*) (2.8 fold and *p* = 0.003), fibroblast growth factor 2 (*FGF2*) (4.7 fold and *p* = 0.03), insulin growth factor 1 receptor (*IGF1R*) (2.0 fold and *p* = 0.01), estrogen receptor 2 (*ESR2*) (6.6 fold and *p* = 0.01), and tumor necrosis factor receptor superfamily member 11A (*TNFRSF11A*) (3.2 fold and *p* = 0.02) (Fig. 4c). Five genes were significantly down-regulated (≥2 fold and *p* ≤ 0.05) in the TC71VR cells compared with parental cells: aryl hydrocarbon receptor nuclear translocator (*ARNT*) (2.1 fold decreased and *p* = 0.01), *CYP2B6* (4.2 fold decreased and *p* = 0.02), *ERBB2* (4.2 fold decreased and *p* = 0.02), *ERBB3* (16.7 fold decreased and *p* = 0.01), and retinoic acid receptor beta (*RARb*) (2.8 fold decreased and *p* = 0.01). Taken together, the VCR-resistant RMS and EWS cell lines that we established showed up-regulation of *GLI1*.

### Modulation of GLI1 activity affects sensitivity of VCR-resistant RMS and EWS cell lines to VCR

**Fig. 6:**
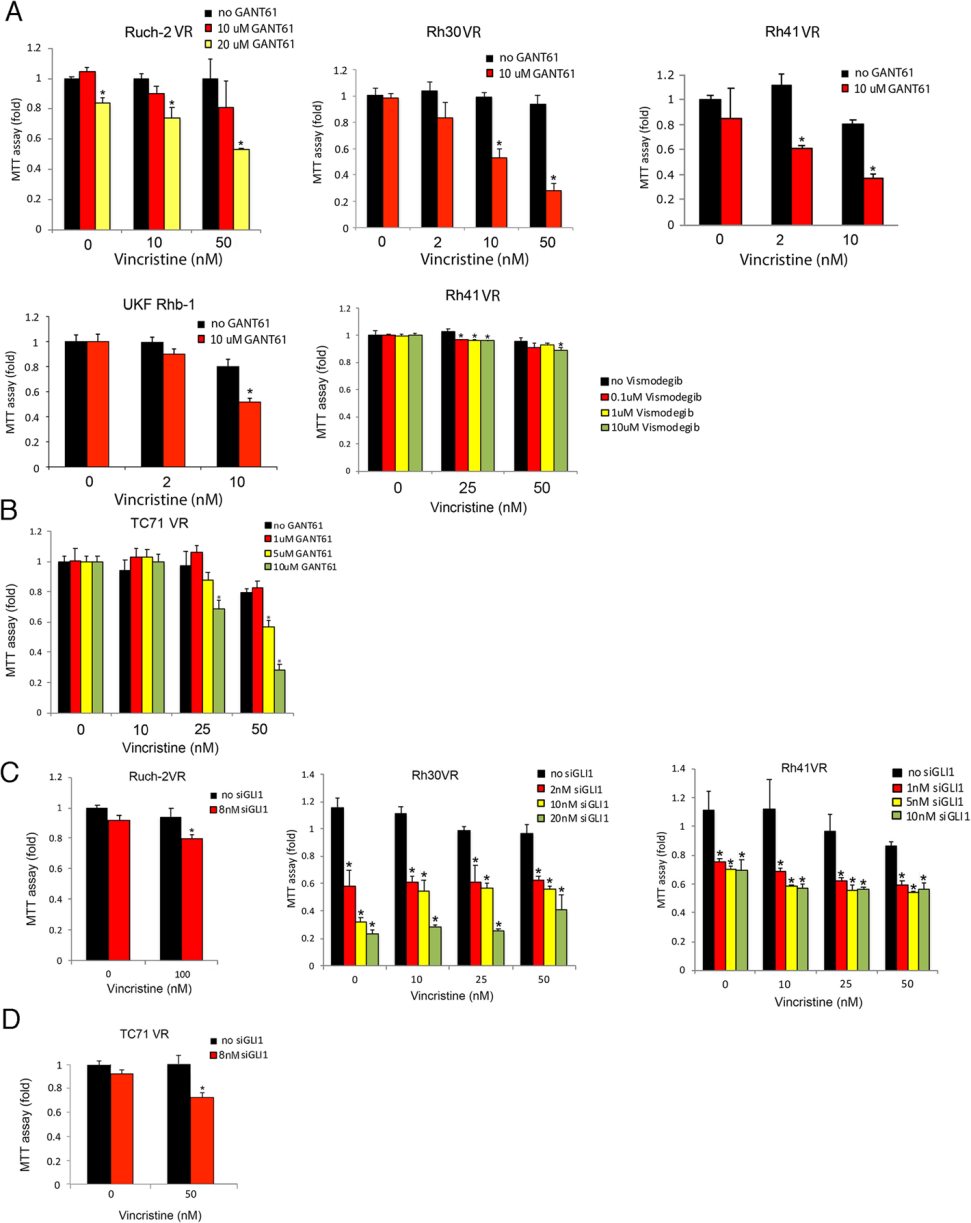
Effect of modulation of GLI1 activity on VCR sensitivity of RMS and EWS cells. **a** GANT61 enhanced sensitivity of VCR-resistant RMS cells to VCR. Vismodegib enhanced sensitivity of Rh41VR cells to VCR to a smaller extent GANT61. The experiments were performed in triplicate and results are expressed as an average with standard deviation. * indicates statistically significant differences compared with treatment with VCR alone (black bars) (*p* ≤ 0.05). **b** GANT61 enhanced sensitivity of VCR-resistant EWS cells to VCR. The experiments were performed in triplicate and results are expressed as an average with standard deviation. * indicates statistically significant differences compared with treatment with VCR alone (black bars) (*p* ≤ 0.05). **c***GLI1* siRNA enhanced sensitivity of Ruch-2VR (top panel) RMS cells to VCR. Rh30VR (middle panel) and Rh41VR (bottom panel) cells were sensitive to *GLI1* siRNA without VCR. The experiments were performed in triplicate and results are expressed as an average with standard deviation. * indicates statistically significant differences compared with VCR alone (black bars) (*p* ≤ 0.05). **d***GLI1* siRNA enhanced sensitivity of TC71VR EWS cells to VCR. The experiments were performed in triplicate and results are expressed as an average with standard deviation. * indicates statistically significant differences compared with VCR alone (black bars) (*p* ≤ 0.05). Microsoft Exel and Adobe Photoshop were used to prepare Figure 6

Next, we tested whether SMO inhibition with vismodgeb or GLI1 inhibition either through pharmacologic inhibition or *GLI1* siRNA, enhanced sensitivity of VCR-resistant RMS and EWS cell lines to treatment with VCR. Treatment of VCR-resistant ARMS or ERMS cells (Ruch-2VR, Rh30VR, Rh41VR, and UKF Rhb-1 cells) or VCR-resistant EWS cells (TC71VR) with VCR together with GANT61 significantly decreased cell viability (MTT assay) at doses that did not reduce cell viability individually (Fig. 6a and b). Treatment of Rh41VR cells with VCR together with vismodegib reduced cell viability to a much smaller extent than seen with GANT61 (Fig. 6a). Treatment of Ruch-2VR ARMS cells and TC71VR EWS cells with VCR together with *GLI1* siRNA also significantly decreased cell viability at doses that did not reduce cell viability individually. However, treatment of Rh30VR and Rh41VR ARMS cells with *GLI1* siRNA alone impacted cell viability even at the lowest concentration we tested (Fig. 6c and d).

In summary, these experiments demonstrate that *GLI1* up-regulation contributes to VCR-resistance of RMS and EWS cell lines and suggest that targeting GLI1 may benefit patients with RMS or EWS by reducing multidrug resistance.

## Discussion

We have shown that ERMS, ARMS, and EWS cell lines express HH pathway components and that most of the cell lines have primary cilia, the organelle in which HH signal transduction occurs. We chose an in vitro approach to assess HH responsiveness and roles for GLI1 in drug resistance using well characterized RMS and EWS cell lines. Some of the cell lines (RD, Rh18, Rh41, CHLA9, CHLA10, TC32, and TC71) showed evidence of HH responsiveness with up-regulation of *GLI1* and/or *PTCH1* through the canonical pathway even when non-canonical mechanisms of activation of *GLI1* may be present, such as EWS cell lines with a EWSR1-FLI1 fusion. This suggests that both canonical and non-canonical mechanisms may lead to *GLI1* expression in RMS and EWS cells and that paracrine HH-signaling may also contribute to the behavior of these tumors in vivo. We saw the largest effects with DHH or SHH for RMS cell lines and with SHH for EWS cell lines. We did not see up-regulation of *GLI1* or *PTCH1* following exposure to HH ligands in Rh30 and CHLA258 cells, both of which have high basal levels of *GLI1* expression. Rh30 RMS cells have *GLI1*-amplification and CHLA258 EWS cells, which were established at the time of recurrence, have a higher level of *GLI1* expression than the other EWS cell lines. Presumably, non-canonical mechanisms drive high constitutive levels of *GLI1* expression in both of these cell lines. Although EWS cell lines that were established at the time of recurrence and presumably have some degree of drug resistance showed higher levels of *GLI1* expression than a EWS cell line established at the time of diagnosis, we could not make a similar comparison for the RMS cell lines. The information we had regarding the RMS cell lines included whether they were established before or after receiving any therapy and not whether they were established before or after recurrence. Previously treated cells could remain sensitive to chemotherapy and may not recur.

We analyzed the role of GLI1 in RMS and EWS cell lines in vitro by up-regulating HH signaling or down-regulating GLI1 activity with the small molecule inhibitor GANT61. It has been previously reported that GANT61 effects in RMS cell lines are specifically mediated through GLI1 inhibition [33]. Although we did not see effects of SHH exposure on cell viability or proliferation, we did see increased apoptosis in RMS and EWS cells and reduced viability and proliferation in EWS cells with GANT61 treatment, suggesting fundamental roles for GLI1 in the biology of these sarcoma cell lines.

Our focus was to determine whether up-regulation of *GLI1* occurs as cells develop resistance to VCR. We chose VCR because it is a P-glycoprotein multi-drug resistance substrate and chemotherapeutic agent that is used for treatment of RMS and EWS [1–4]. We established VCR-resistant RMS and EWS cell lines, which we defined as an increase in the IC_50_ of ≥30 fold. We were able to achieve this in four (Ruch-2VR, Rh30VR, Rh41VR, and TC71VR cells) of ten cell lines that we attempted. We focused our analysis of the drug resistance array results in RMS cell lines on genes whose expression was altered in the same direction in resistant cells compared with the corresponding parental cells in more than one cell line. We were only able to establish a single VCR-resistant EWS cell line, which limits conclusions that can be made in EWS.

We saw up-regulation of *GLI1* in all of the VCR-resistant cells by RT PCR array, although the magnitude of change did not reach the bar of 2.0-fold and *p* ≤ 0.05 for Ruch-2VR ERMS cells (1.7 fold and *p* = 0.02). The only other gene in the 86-gene drug resistance array that was up-regulated in Rh30VR, Rh41VR, and TC71VR cells was the known direct GLI1 target and multidrug resistance gene, *MDR1*. Only one gene was down-regulated in Rh30VR, Rh41VR, and TC71VR cells, the receptor tyrosine kinase, *ERBB3*. *ERBB3*, a member of the epidermal growth factor receptor family, is often aberrantly expressed and/or activated in cancers and has been associated with drug resistance [47]. It is unclear why this receptor tyrosine kinase is down-regulated in the VCR-resistant RMS and EWS cells compared with their parental cells. Curiously, *ERBB3* localizes to 12q13, the same genomic region as *GLI1*, raising the possibility of complex co-regulation. Based on up-regulation of *MVP* in both VCR-resistant ARMS cell lines (Rh30VR and Rh41VR), we demonstrated that *MVP* is a direct transcriptional target of GLI1 using gel mobility shift assays, chromatin immunoprecipitation, and cotransfection assays. Down-regulation of *CYP1A1* and *CYP2D6* was also seen in both of the ARMS cell lines (Rh30VR and Rh41VR) possibly based on a decreased need for detoxifying enzymes in the presence of active drug efflux pumps.

We believe that canonical and non-canonical mechanisms contribute to *GLI1* up-regulation and therefore focused on the effect of GLI1 inhibition rather than SMO inhibition on VCR sensitivity. In support of this decision, the GLI1 inhibitor GANT61 reversed VCR resistance more effectively in Rh41VR cells than the SMO inhibitor vismodegib. We were able to show that *GLI1* down-regulation, either by GANT61 or *GLI1* siRNA, in the VCR-resistant cell lines enhanced their sensitivity to VCR in vitro, supporting a role for GLI1 in RMS and EWS VCR resistance. Although statistically significant, the magnitude of the effect was somewhat limited when using our experimental conditions for *GLI1* siRNA in Ruch-2VR and TC71VR cells. Rh30VR and Rh41VR cells were sensitive to *GLI1* siRNA treatment even at the lowest concentrations tested, suggesting that viability of these cell lines is dependent on GLI1. This work supports the importance of developing ways to inhibit the GLI1 transcription factor, which may include targeting *GLI1* expression or GLI1-coactivator interactions [48, 49].

## Conclusions

In this study we showed that RMS and EWS cell lines express HH pathway components and are often responsive to HH ligands, with up-regulation of *GLI1* and/or *PTCH1* expression. HH signaling appears to play roles in cell survival in RMS and cell survival as well as proliferation in EWS cells. Up-regulation of *GLI1* expression occurred in most cells as they developed resistance to VCR. In addition to *MDR1*, the ATP-binding cassette family member *MVP* is also a direct target of GLI1 and can also contribute to VCR resistance. Treatment of vincristine-resistant cells with the GLI1 small molecule inhibitor GANT61 or *GLI1* siRNA together with VCR significantly decreased cell viability at doses that did not reduce viability individually. These experiments demonstrate that *GLI1* up-regulation contributes to VCR resistance in RMS and EWS cell lines and suggest that targeting GLI1 may benefit patients with RMS or EWS by reducing multidrug resistance.

## Supplementary information

**Additional file 1.**

**Additional file 2.**

**Additional file 3.**

**Additional file 4.**

## Data Availability

The datasets used and/or analyzed during this study are available from the corresponding author on reasonable request.

## Abbreviations

DAPI: 4′,6-diamidino-2-phenyindole
BrdU: 5-bromo-2′-deoxyuridine
ARMS: Alveolar rhabdomyosarcoma
ARNT: Aryl hydrocarbon receptor nuclear translocator
MDR1: ATP-binding cassette sub-family B member 1
TAP1: ATP binding cassette subfamily B member, transporter 1
ChIP: Chromatin immunoprecipitation
CTR: Control cells
CCNE1: Cyclin E1
DHH: Desert hedgehog
ERMS: Embryonal rhabdomyosarcoma
ESR2: Estrogen receptor 2
EWS: Ewing sarcoma
FGF2: Fibroblast growth factor 2
HH: Hedgehog
IHH: Indian hedgehog
IGF1R: Insulin growth factor 1 receptor
kD: Kilodaltons
MVP: Major vault protein
MTT: Methylthiazolyl diphenyl-tetrazolium bromide
PTCH: Patched
qRT PCR: Quantitative reverse transcriptase polymerase chain reaction
RARb: Retinoic acid receptor beta
RT PCR: Reverse transcriptase polymerase chain reaction
RMS: Rhabdomyosarcoma
SMO: Smoothened
SHH: Sonic hedgehog
SUFU: Suppressor of Fused
Ct: Threshold cycle
TNFRSF11A: Tumor necrosis factor receptor superfamily member 11A
VCR: Vincristine
