# Supplementary figures and images for: Up-regulation of GLI1 in vincristine-resistant rhabdomyosarcoma and Ewing sarcoma
Source: BMC Cancer. 2020 Jun 3;20:511. doi: 10.1186/s12885-020-06985-0 (PMC7310145; doi:10.1186/s12885-020-06985-0)

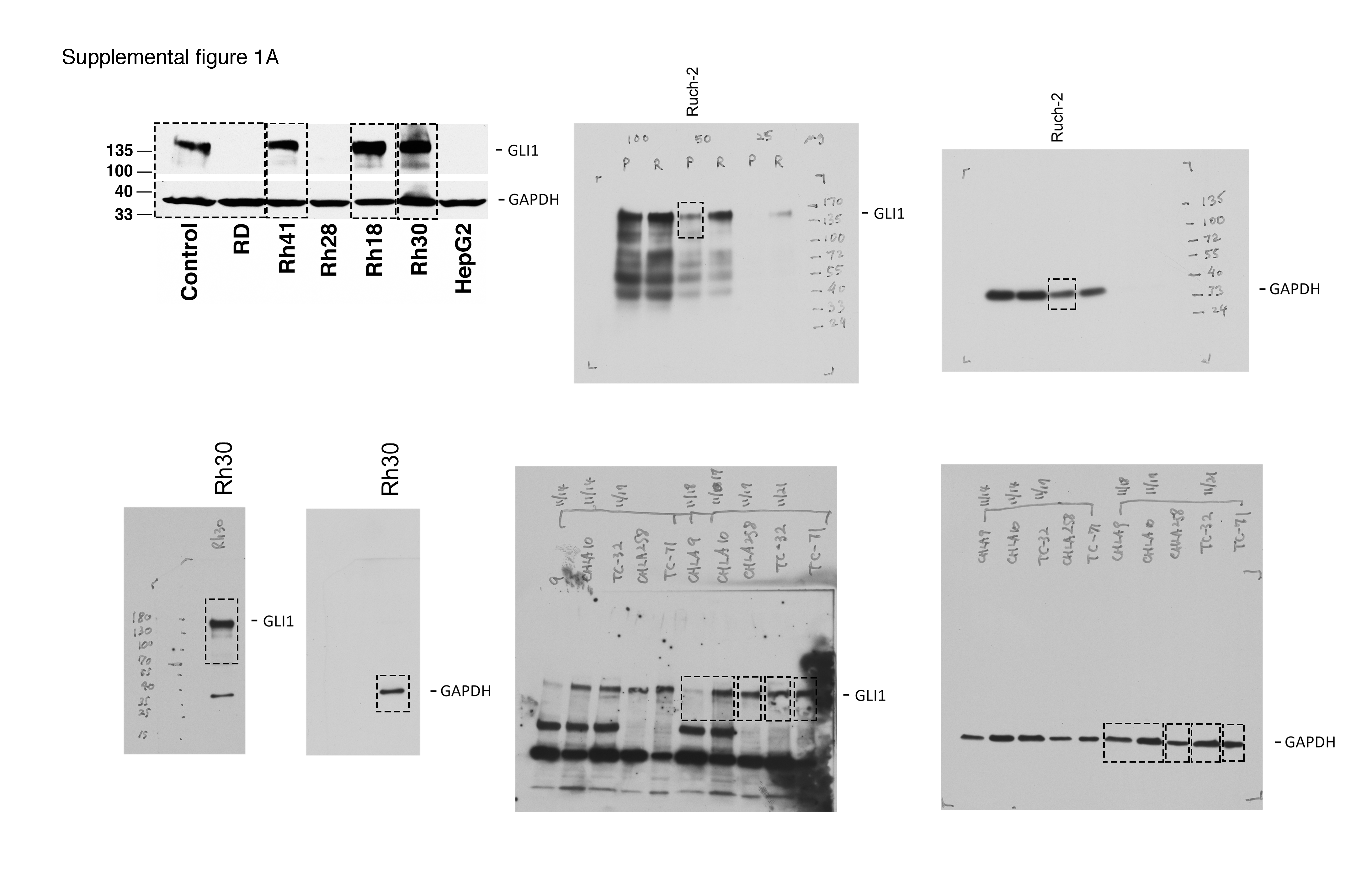

Supplement: Supplementary file 1 — Additional file 1. [file 12885_2020_6985_MOESM1_ESM.tif]

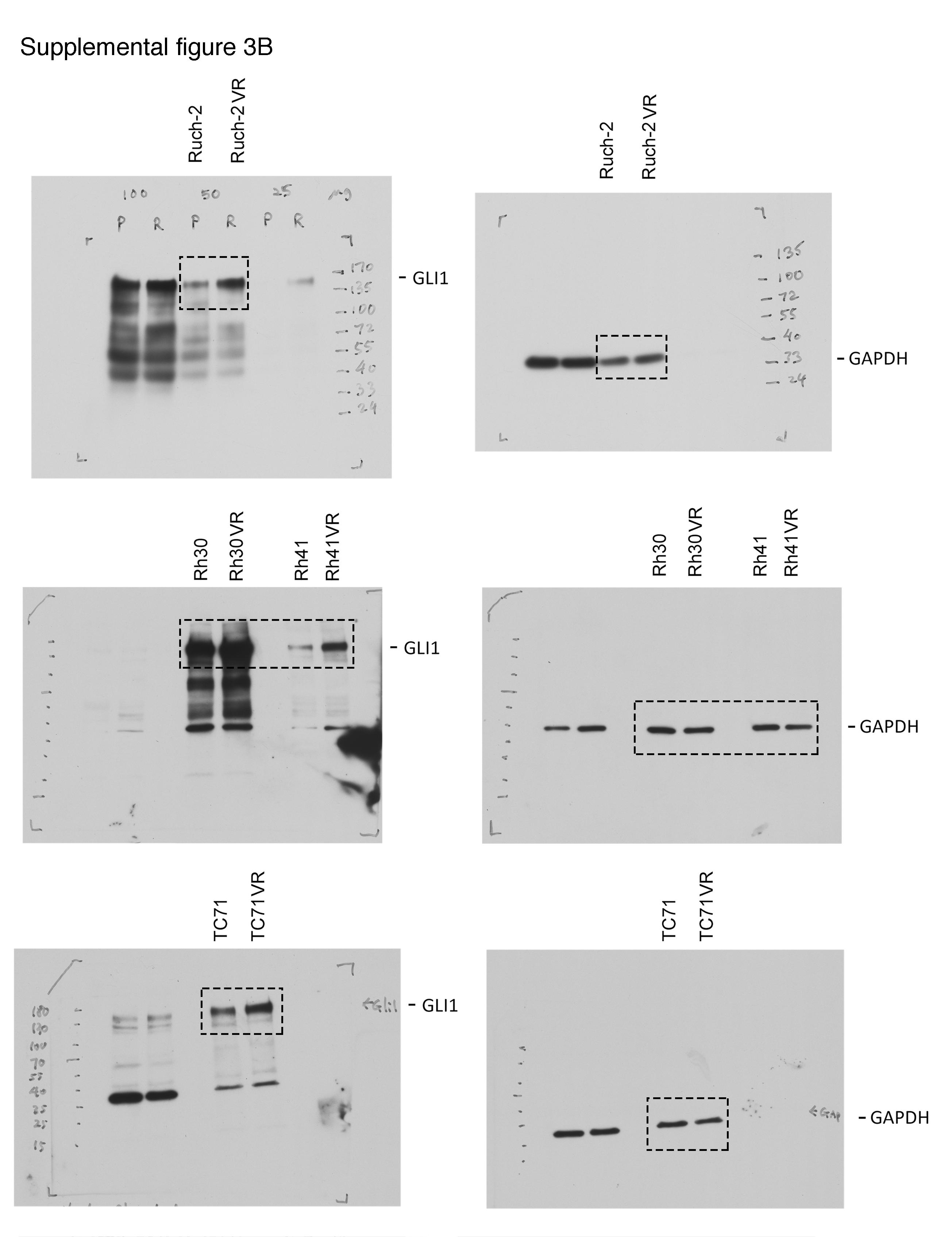

Supplement: Supplementary file 2 — Additional file 2. [file 12885_2020_6985_MOESM2_ESM.tif]

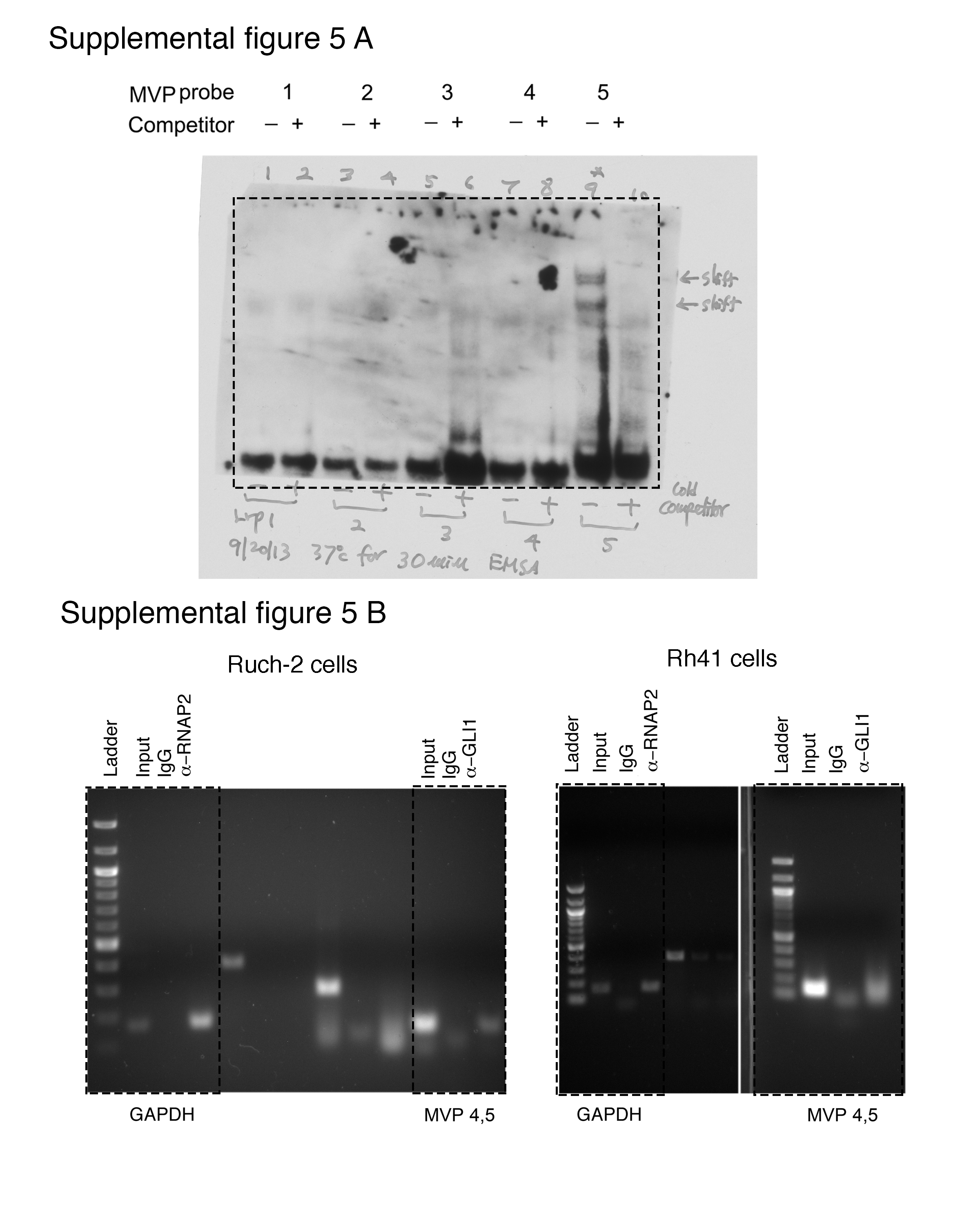

Supplement: Supplementary file 3 — Additional file 3. [file 12885_2020_6985_MOESM3_ESM.tif]
